# Mixed effect analysis of factors influencing the use of insecticides treated bed nets among pregnant women in Ghana: evidence from the 2019 Malaria Indicator Survey

**DOI:** 10.1186/s12884-022-04586-2

**Published:** 2022-03-27

**Authors:** Desmond Klu, Matilda Aberese-Ako, Alfred Kwesi Manyeh, Mustapha Immurana, Phidelia Doegah, Maxwell Dalaba, Evelyn Acquah, Robert Kaba Alhassan, Evelyn Korkor Ansah

**Affiliations:** 1 Centre for Malaria Research, Institute of Health Research, University of Health and Allied Sciences, Ho, Ghana; 2 Centre for Neglected Tropical Disease Research, Institute of Health Research, University of Health and Allied Sciences, Ho, Ghana; 3 Centre for Non-Communicable Disease Research, Institute of Health Research, University of Health and Allied Sciences, Ho, Ghana; 4 Centre for Health Policy and Implementation Research, Institute of Health Research, University of Health and Allied Sciences, Ho, Ghana

**Keywords:** Malaria, Pregnant women, ITN, Prevention, Ghana

## Abstract

**Background:**

Malaria during pregnancy is a major cause of maternal morbidity globally and leads to poor birth outcomes. The World Health Organization has recommended the use of insecticide treated bed nets (ITN) as one of the effective malaria preventive strategies among pregnant women in malaria endemic areas. This study, therefore, seeks to examine the individual and household factors associated with the use of ITNs among pregnant women in Ghana.

**Methods:**

Data for this study was obtained from the 2019 Ghana Malaria Indicator Survey (GMIS) conducted between September 25 and November 24, 2019. The weighted sample comprised 353 pregnant women aged 15–49 years. Data was analyzed with SPSS version 22 using both descriptive and multilevel logistics regression modelling. Statistically significant level was set at *p* < 0.05.

**Results:**

The study found that 49.2% of pregnant women in Ghana use ITN to prevent malaria. Pregnant women aged 35–49 years (AOR = 3.403, CI: 1.191–9.725), those with no formal education (AOR = 5.585, CI = 1.315–23.716), and those who had secondary education (AOR = 3.509, CI = 1.076–11.440) had higher odds of using ITN. Similarly, higher odds of ITN usage was found among who belonged to the Akan ethnic group (AOR = 7.234, CI = 1.497–34.955), dwell in male-headed households (AOR = 2.232, CI = 1.105–4.508) and those whose household heads are aged 60–69 years (AOR = 4.303, CI = 1.160–15.966). However, pregnant women who resided in urban areas (AOR = 0.355, CI = 0.216–0.582), those whose household heads aged 40–49 years (AOR = 0.175, CI = 0.066–0.467) and those who belonged to richer (AOR =0.184, CI = 0.050–0.679) and richest (AOR = 0.107, CI = 0.021–0.552) households had lower odds of using ITN for malaria prevention.

**Conclusions:**

Individual socio-demographic and household factors such as pregnant women’s age, educational level, place of residence, ethnicity, sex and age of household head, and household wealth quintile are associated with the use of ITN for malaria prevention among pregnant women. These factors ought to be considered in strengthening malaria prevention campaigns and develop new interventions to help increase ITN utilization among vulnerable population living in malaria- endemic areas.

## Background

Malaria as a vector borne disease is still a global health problem especially in sub-Saharan Africa and the most adversely affected are pregnant women and children under five [1, 2]. According to the World Health Organisation (WHO), in 2019, an estimated 11 million pregnant women were exposed to malaria infections. These pregnant women delivered 872,000 children with low birth weight, with West Africa having the highest prevalence of low birth weight children due to malaria in pregnancy [1]. Further, an estimated 25 million pregnant women are currently at risk of malaria infection. Meanwhile, malaria infection among pregnant women account for over 10,000 maternal and 200,000 neonatal deaths per annum [3, 4]. Malaria in pregnancy is associated with risk of miscarriages, stillbirths and, intrauterine demise [5–7].

In Ghana, malaria cases recorded at Out-patient Departments (OPD) in 2017 was 399,736 compared to 383,034 in 2016 [8] among pregnant women. This figure represents an increase of 4.2% over the 2016-recorded number of cases among pregnant women. To help reduce Malaria in Pregnancy (MIP), the National Malaria Control Programme (NMCP) has introduced a number of preventive interventions such as Intermittent Preventive Treatment of malaria in Pregnancy (IPTp), distribution and use of Insecticide Treated Nets (ITNs) and Indoor Residual Spraying (IRS) [9–11]. Other malaria prevention strategies for vulnerable population are Seasonal Malaria Chemoprevention (SMC), and Integrated Community Case Management (iCCM) [11].

Among these interventions, available empirical evidence suggests that the use of ITN is a cost-effective vector control measure for the prevention of malaria transmission especially in highly endemic areas [12–17]. The appropriate and effective use of ITNs has been shown to decrease the transmission of malaria by 90% [18] and negative pregnancy outcomes by 33% [18, 19]. It is therefore not farfetched that WHO recommends the use of ITN as an effective measure for the prevention of malaria in pregnancy [1, 2].

However, the effective and continuous use of ITN by pregnant women in the African sub-region including Ghana has been relatively low [20–24]. A number of reasons and factors have been associated with the low utilization of ITN. Some of these reasons and factors include discomfort with sleeping under ITNs [20, 25–27], poor knowledge and perception of ITNs [28, 29], high cost of ITNs [30], distance to the nearest health facility [31, 32], age of pregnant women, area of residence, educational level, marital status, and wealth index [33–40].

In Ghana, ITN utilization among pregnant women decreased from 59% in 2016 to 49% in 2019 [11], implying that Ghana still has a long way to go in its efforts to achieve universal coverage of ITNs. The universal coverage of ITNs has been defined as ITN use by over 80% of vulnerable populations in malaria endemic areas to achieve optimum protection [41]. Although previous studies have examined factors affecting ITN utilization among vulnerable sub-populations such as pregnant women across different geographical locations including Ghana [26, 30, 34, 36, 39], to the best of our knowledge none of the studies have focused on how individual and household factors interact to influence the use of ITNs among pregnant women. This paper, therefore, seeks to examine the mixed effects of individual and household factors on the use of ITNs among pregnant women in Ghana using the 2019 Ghana Malaria Indicator Survey (GMIS).

## Materials and methods

### Data source

The data for this study was (secondary data) extracted from the 2019 Ghana Malaria Indicator Surveys (GMIS) which was conducted from September 25 to November 24, 2019. We used data from the women’s file (15–49 years). The GMIS collects information on malaria prevention (ownership and use of treated mosquito bed nets and assess coverage of intermittent preventive treatment to protect pregnant women against malaria), treatment, and prevalence in Ghana. In this study, data on a weighted sub-sample of women who reported they were pregnant during the surveys was extracted and analyzed.

### Survey and participants

Details concerning the scope and methodology of the GMIS have already been published [11]. Briefly, the nationally representative survey was implemented by the Ghana Statistical Service (GSS), Ministry of Health (MOH) and National Malaria Control Programme of the Ghana Health Service with technical support from Inner City Fund (ICF) through the Demographic and Health Surveys (DHS) Program.

### Sampling and sample size

The total number of women within the reproductive ages 15–49 years in the 2019 GMIS was 5181. However, in this study we limited the analysis to women who reported being pregnant during the survey. We weighted the entire data before sampling out pregnant women in all the 16 regions of Ghana. Therefore, the weighted sample of respondents (currently pregnant women) in the 2019 GMIS was 353.

### Study variables

#### Dependent variable

The dependent variable for this study was the use of ITN. An ITN was defined in this study as a bed net that has been treated with insecticide to protect against mosquito bites and malaria. Eligible women were asked whether they slept under a treated mosquito net the night prior to the survey or otherwise. We coded ‘1’ for pregnant women who indicated they slept under ITN and coded ‘0’ for those who did not.

#### Predictor variables

We considered both individual and household level factors in this study. The rationale for the choice of these factors was based on their statistically significant association with ITN utilization in earlier studies [26, 29, 32, 35].

#### Individual socio-demographic factors

The individual-level socio-demographic factors included age of pregnant women (15–24, 25–34, 35–49) educational level (no education, primary, secondary, higher) religion (catholic, protestant, Muslim, pentecostal/charismatic, no religion) literacy level (illiterate, literate), ecological zones of residence (coastal zone, middle belt, northern zone) place of residence (urban, rural) ethnicity (Akan, Ga/Dangme, Ewe, Mole-Dagbani, Others) and parity (1–3, 4–6, 7 or more).

#### Household level factors

We considered the following household-level factors in the study: sex of household head (male and female), age of household head (20–29,30–39, 40–49, 50–59,60–69, 70+) and household wealth quintile (poorest, poorer, middle, richer, richest). The other variables included household source of drinking water, type of toilet facility and type of cooking fuel used by the household. The measurement and classification of the variable ‘*household source of drinking water’* was guided by the WHO/United Nations International Children’s Emergency Fund Joint Monitoring Programme for Water Supply, Sanitation and Hygiene (WHO/UNICEF-JMP) classification of source of drinking water. For this study, the variable had been classified into two: improved and unimproved source of drinking water. In this study, improved source of drinking water comprised pipe-borne water inside dwelling, piped into dwelling, pipe to yard/plot, piped to neighbour’s house/compound, tube well water, borehole, protected dug well, protected well, protected spring and rainwater collection, bottled water and sachet water. Unimproved source of drinking water in this study included unprotected well, surface from spring, unprotected spring, river/dam, tanker truck and cart with a small tank. Type of toilet facility was also categorized into two improved and unimproved. The classification of improved toilet facility was also guided by the WHO/UNICEF-JMP classification of sanitation technologies. Improved toilet facilities in this study comprised flushed to pipe sewer, flushed to septic tank, flushed to pit latrine, flushed to unknown place, flushed to bio-digester, ventilated improved pit latrine (VIP), pit latrine with slab, pit toilet latrine and composting toilets. The unimproved toilet facility included flush to somewhere else, pit without slab/open pit, no facility, bush/field and hanging toilet/latrine. Type of household cooking fuel was categorized into the following: Liquified Petroleum Gas (LPG), Charcoal, Fuel wood and other cooking fuel (straw/shrub/grass, agricultural crops, and animal dung).

### Statistical analysis

We analyzed the data with SPSS version 22. In analyzing the data, we followed three stages. The first stage was the use of simple descriptive statistics to describe the outcome and predictor variables. The second stage involved a cross-tabulation of all the individual and household level factors against the use of ITN among pregnant women. In the third stage, we developed three different models that involved multilevel binary logistic regression analyses to assess the effect of individual and household level factors on pregnant women’s use of insecticides treated bed nets. Model I analyzed the effect of only individual-level factors, model II analyzed the effect of only household-level factors, while model III analyzed both individual and household level factors on the use of ITN among pregnant women. For all three models, we presented the adjusted odds ratios (AOR) and their associated 95% confidence intervals (CIs). We also applied sample weight (v005/1,000,000) in weighting the entire data to correct possible over and under sampling issues.

## Results

### Socio-demographic and insecticide-treated bed net utilization among pregnant women in Ghana

Table 1 shows the individual socio-demographic characteristics of ownership and use of ITN among pregnant women in Ghana. About 85% of pregnant women owned at least one treated bed net in Ghana. A higher proportion (50.8%) of pregnant women reported not using treated bed nets compared to 49.2% who used ITNs. About 46% of the respondents were aged 25–34 years constituting the highest proportion of pregnant women in any of the age categories. More than half (51.6%) had attained secondary level of education and an almost equal proportion were literate. With regards to residence, a higher proportion (40.8%) of pregnant women resided in the middle belt compared to other ecological zones of the country. More than half (58.5%) of pregnant women resided in rural areas with a higher proportion (38.9%) belonging to the Akan ethnic group. About 7 out of 10 pregnant women in Ghana had between 1 and 3 children while 5% had 7 or more children.

**Table 1:** Individual socio-demographic characteristics and use of ITN net among pregnant women in Ghana

Variables	Weighted Sample ***n*** = 353	%
**Ownership of treated bed net**		
Do not own	54	15.4
Own	299	84.6
**Use of mosquito treated bed net**		
Do not use bed net	179	50.8
Use treated bed net	175	49.2
**Socio-Demographic Factors**		
**Age**		
15–24	123	34.8
25–34	162	46.0
35–49	68	19.2
**Education Level**		
No education	72	20.3
Primary	74	21.0
Secondary	182	51.6
Higher	25	7.2
**Religion**		
Catholic	31	8.8
Protestant	32	9.1
Moslem	81	22.8
Pentecostal/Charismatic	193	54.6
No Religion	16	4.6
**Literacy Level**		
Illiterate	172	48.8
Literate	181	51.2
**Ecological zones of residence**		
Coastal Zone	136	38.6
Middle Belt	144	40.8
Northern Zone	73	20.6
**Place of Residence**		
Urban	147	41.5
Rural	207	58.5
**Ethnicity**		
Akan	138	38.9
Ga/Dangme	22	6.3
Ewe	67	19.1
Mole-Dagbani	111	31.3
Other	15	4.3
**Parity**		
1–3	235	66.7
4–6	101	28.5
7+	17	4.8

Source: Computed from 2019 Ghana Malaria Indicator Surveys (GMIS)

### Household factors and use of insecticide treated bet net among pregnant women in Ghana

Table 2 shows the household characteristics of pregnant women and their utilization of ITN. The results showed that 77.1% of pregnant women belonged to male-headed households. The highest proportion (35.4%) of the heads of households were between ages 30–39 years. Highest proportion of (22.0%) pregnant women belonged to the poorest household wealth quintile category with the lowest proportion (18.9%) belonging to the richer wealth quintile. About 6 out of 10 pregnant women belong to households that access improved source of drinking water as well as improved toilet facility. Additionally, 44.4% of pregnant women in 2019 belonged to households that used fuel wood as the main type of cooking fuel. This proportion constituted the highest compared to other types of household cooking fuel.

**Table 2:** Household characteristics and use of ITN among pregnant women in Ghana

Household Level factors	Weighted Sample *n* = 353	%
**Sex of Household Head**		
Male	272	77.1
Female	81	22.9
**Age of Household Head**		
20–29	57	16.2
30–39	125	35.4
40–49	83	23.6
50–59	44	12.4
60–69	25	7.1
70+	19	5.3
**Household Wealth Quintile**		
Poorest	78	22.0
Poorer	74	21.0
Middle	69	19.4
Richer	66	18.6
Richest	67	18.9
**Household source of drinking water**		
Improved source of drinking water	210	59.5
Unimproved source of drinking water	143	40.5
**Household type of toilet facility**		
Improved toilet facility	234	66.1
Unimproved toilet facility	120	33.9
**Household type of cooking fuel**		
Liquefied Petroleum Gas (LPG)	81	22.9
Charcoal	103	29.3
Fuel wood	157	44.4
Other cooking fuel	12	3.4

Source: Computed from 2019 Ghana Malaria Indicator Survey (GMIS)

### Association between individual and household level factors and use of ITN among pregnant women in Ghana

Table 3 shows a chi-square analysis between individual-level and household level factors and use of ITN among pregnant women in Ghana. Individual socio-demographic factors of pregnant women such as educational level, literacy level, ecological zone of residence, place of residence, ethnicity and parity were found to be significantly associated with the use of ITN at *p* < 0.05. With regards to household level factors, sex of household head, age of household head, wealth quintile, household source of drinking water, type of toilet facility and type of cooking fuel had a significant association with use of ITN among pregnant women in Ghana at *p* < 0.05.

**Table 3:** Association between individual, household level factors and use of ITN among pregnant women in Ghana

Factors	Pregnant women use of insecticide treated bed nets		
	Not Using	Using	*P*-values
**Individual Level Factors**			
**Age**			
15–24	54.5	45.5	0.192
25–34	52.1	47.9	
35–49	41.2	58.8	
**Educational Level**			
No Education	36.6	63.4	0.002**
Primary	51.4	48.6	
Secondary	52.2	47.8	
Higher	80.0	20.0	
**Religion**			
Catholic	37.5	62.5	0.141
Protestant	37.5	62.5	
Moslem	50.6	49.4	
Pentecostal/Charismatic	56.0	44.0	
No Religion	43.8	56.3	
**Literacy level**			
Illiterate	43.4	56.6	0.007**
Literate	57.8	42.2	
**Ecological Zone**			
Coastal Zone	59.1	40.9	0.005**
Middle Belt	50.7	49.3	
Northern Zone	35.6	64.4	
**Place of Residence**			
Urban	67.3	32.7	0.000***
Rural	39.1	60.9	
**Ethnicity**			
Akan	49.3	50.7	0.012*
Ga/Dangme	69.6	30.4	
Ewe	58.8	41.2	
Mole-Dagbani	40.5	59.5	
Other	73.3	26.7	
**Parity**			
1–3	55.7	44.3	0.034*
4–6	41.6	58.4	
7+	38.9	61.1	
**Household Factors**			
**Sex of Household Head**			
Male	47.4	52.6	0.024*
Female	61.7	38.3	
**Age of Household Head**			
20–29	52.6	47.4	0.002**
30–39	53.6	46.4	
40–49	56.6	43.4	
50–59	55.8	44.2	
60–69	16.0	84.0	
70+	36.8	63.2	
**Wealth Index**			
Poorest	29.5	70.5	0.000***
Poorer	31.1	68.9	
Middle	52.2	47.8	
Richer	66.7	33.3	
Richest	80.6	19.4	
**Source of drinking water**			
Improved	44.8	55.2	0.005**
Unimproved	60.1	39.9	
**Type of toilet facility**			
Improved	60.1	39.9	0.000***
Unimproved	32.5	67.5	
**Type of cooking fuel**			
Liquefied Petroleum Gas	80.2	19.8	0.000***
Charcoal	60.2	39.8	
Fuel wood	30.1	69.9	
Other cooking fuel	41.7	58.3	

Source: Computed from 2019 Ghana Malaria Indicator Surveys (GMIS) *p<0.05; **p<0.01; ***p<0.001

There was a significant difference [*p* = 0.002] in ITN use among pregnant women by their educational level with higher ITN use being recorded among women with no education (63.4%) relative to the other categories. Similarly, higher use of ITN was found among illiterate pregnant women (56.6%) compared to literate pregnant women (42.2%) at *p* = 0.007. The study also found that, a higher proportion (64.4%) of pregnant women who reside in the Northern ecological zone used ITNs more as compared to those who reside in the Middle belt (49.3%) and Coastal zone (40.9%) [*p* = 0.005]. There was also a significant difference in ITN use among pregnant women who are urban residents (32.7%) and those who resided in rural areas (60.9%) [*p* < 0.001]. Pregnant women who belong to the Mole-Dagbani ethnic background recorded higher use (59.5%) of ITN relative to other ethnic groups (*p* = 0.012). Higher use of ITN was recorded among pregnant women with 7 or more children (61.1%) followed by those with 4–6 children and 1–3 children represented by 58.4 and 44.3% respectively (*p* = 0.034).

Regarding household factors, a statistically significant [*p* = 0.024] difference was established between the use of ITN among pregnant women who dwell in male-headed (52.6%) households and those who dwell in female-headed households (38.3%). Pregnant women who resided in households headed by persons aged 60–69 years (84.0%) recorded higher ITN utilisation compared to other age categories [*p* = 0.002]. ITN use by pregnant women was higher among pregnant women from the poorest household wealth quintiles (70.5%) as compared to those from the richest household quintiles (19.4%) [*p* < 0.001]. A statistically significant association [*p* = 0.005] was found between household source of drinking water and ITN utilisation, with higher ITN use recorded among pregnant women who belong to household that have access to improved sources of drinking water (55.2%) compared to those using unimproved drinking water sources (39.9%). A higher proportion of pregnant women belonging to households with unimproved toilet facilities (67.5%) used ITN relative to their counterparts who had access to improved toilet facilities (39.9%). ITN use was higher among pregnant women who belonged to the household that used fuel wood (69.9%) as their main source of cooking fuel than among those from households that used other types of household cooking fuels [*p* = 0.000].

### Individual and household factors influencing the use of ITN among pregnant women in Ghana

Table 4 presents results on the individual and household factors that influence the use of ITN among pregnant women in Ghana. In the Model 1 (only individual-level variables), educational level and place of residence significantly predicted the use of ITN among pregnant women in Ghana. In terms of educational level, the results showed that pregnant women with no formal education (AOR = 5.585, CI: 1.32–23.72) and secondary education (AOR = 3.509, CI: 1.08–11.44) had higher odds of ITN utilisation compared to those with tertiary/higher education. Regarding place of residence, compared to those who resided in rural areas, pregnant women who resided in urban areas (AOR = 0.355, CI: 0.22–0.58) had lower odds of ITN utilisation. In the second model (only household-level variables), sex and age of household heads were significant predictors of ITN use among pregnant women in Ghana. Pregnant women who belonged to male-headed households (AOR = 1.870, CI: 1.00–3.49) had higher likelihood of ITN utilisation relative to those who belong to female headed households. Pregnant women whose household heads were aged between 40 and 49 years were less likely (AOR = 0.437, CI: 0.19–0.99) to use ITN as compared to those whose household heads were aged 20–29 years. Additionally, pregnant women who belonged to households with heads of households aged between 60 and 69 years were more likely to use ITNs as compared to pregnant women whose household heads were aged between 20 and 29 years (AOR = 4.303, CI: 1.16–15.97). The third model, which combined individual and household level factors, showed that age of pregnant women, their ethnic background, sex and age of household heads and household wealth quintile were significant in predicting ITN utilisation among pregnant women in Ghana. Pregnant women aged 35–49 years (AOR = 3.403, CI: 1.19–9.73) had a higher likelihood of using ITN compared to those aged 15–24 years. Pregnant women from Akan ethnic background, were more likely (AOR = 7.234, CI:1.50–34.96) to use ITN as compared to those who belonged to other ethnic groups. Additionally, pregnant women who belonged to households with household heads aged between 40 and 49 years and 50–59 years (AOR = 0.239, CI:0.08–0.72) were less likely to use ITN (AOR = 0.175, CI:0.07–0.47) relative to those whose household heads were aged between 20 and 29 years. Pregnant women from households belonging to the richer wealth quintile (AOR = 0.184, CI: 0.05–0.68) and richest wealth quintiles (AOR = 0.107, CI: 0.02–0.55) were less likely to use ITNs as, compared to those from households belonging to the poorest household wealth index.

**Table 4:** Multilevel logistic regression of individual and household level factors influencing use of ITN among pregnant women in Ghana

Variables	Model I AOR [95%CI]	Model II AOR[95% CI]	Model III AOR [95% CI]
**Individual level factors**			
**Age**			
15–24	Ref		Ref
25–34	1.028 [0.572–1.845]		1.519 [0.721–3.199]
35–49	1.606 [0.693–3.721]		3.403*[1.191–9.725]
**Educational Level**			
No Education	5.585*[1.315–23.716]		1.627 [0.295–8.962]
Primary	3.228 [0.826–12.611]		0.988 [0.191–5.112]
Secondary	3.509*[1.076–11.440]		1.443 [0.356–5.845]
Higher	Ref		Ref
**Religion**			
Catholic	1.435 [0.364–5.658]		1.473 [0.318–6.811]
Protestant	1.472 [0.363–5.971]		1.324 [0.279–6.271]
Moslem	0.665 [0.188–2.348]		0.647 [0.166–2.513]
Pentecostal/Charismatic	0.866 [0.256–2.927]		0.614 [0.159–2.366]
No Religion	Ref		Ref
**Literacy level**			
Illiterate	Ref		Ref
Literate	1.024 [0.522–2.008]		1.389 [0.616–3.133]
**Ecological Zone**			
Coastal Zone	Ref		Ref
Middle Belt	1.336 [0.776–2.300]		1.088 [0.562–2.107]
Northern Zone	2.108 [0.930–4.775]		1.204 [0.416–3.483]
**Place of Residence**			
Urban	0.355***[0.216–0.582]		0.895 [0.450–1.778]
Rural	Ref		Ref
**Ethnicity**			
Akan	2.888 [0.711–11.722]		7.234*[1.497–34.955]
Ga/Dangme	1.224 [0.227–6.612]		2.157 [0.334–13.923]
Ewe	1.524 [0.373–6.224]		3.106 [0.642–15.037]
Mole-Dagbani	2.452 [0.649–9.266]		2.663 [0.593–11.962]
Other	Ref		Ref
**Parity**			
1–3	Ref		Ref
4–6	1.031 [0.538–1.974]		1.549 [0.726–3.302]
7+	0.685 [0.196–2.392]		0.701 [0.166–2.955]
**Household level factors**			
**Sex of Household Head**			
Male		1.870*[1.003–3.486]	2.232*[1.105–4.508]
Female		Ref	Ref
**Age of Household Head**			
20–29		Ref	Ref
30–39		0.683 [0.318–1.467]	0.435 [0.177–1.066]
40–49		0.437*[0.193–0.991]	0.175***[0.066–0.467]
50–59		0.464 [0.181–1.188]	0.239*[0.080–0.716]
60–69		4.303*[1.160–15.966]	4.084 [0.949–17.583]
70+		0.843 [0.241–2.943]	0.743 [0.192–2.875]
**Household Wealth Index**			
Poorest		Ref	Ref
Poorer		1.514 [0.691–3.313]	1.046 [0.396–2.765]
Middle		0.749 [0.304–1.844]	0.449 [0.144–1.400]
Richer		0.509 [0.188–1.373]	0.184*[0.050–0.679]
Richest		0.428 [0.124–1.478]	0.107**[0.021–0.552]
**Household Source of drinking water**			
Improved		Ref	Ref
Unimproved		1.019 [0.586–1.774]	1.314 [0.697–2.479]
**Household Type of toilet facility**			
Improved toilet facility		Ref	Ref
Unimproved toilet facility		1.610 [0.881–2.944]	1.413 [0.725–2.755]
**Household Type of cooking fuel**			
Liquefied Petroleum Gas (LPG)		0.479 [0.099–2.319]	0.479 [0.099–2.319]
Charcoal		0.947 [0.197–4.541]	0.947 [0.197–4.541]
Fuel wood		1.328 [0.282–6.250]	1.328 [0.282–6.250]
Other cooking fuel		Ref	Ref

Source: Computed from 2019 Ghana Malaria Indicator Survey (GMIS)

^*^P < 0.05, ^**^P < 0.01, ***P < 0.001; AOR = Adjusted Odds Ratio

Model I = individual socio-demographic variables

Model II = household variables

Model III = final model adjusted for individual and household level variables

## Discussion

### Summary of main findings

The study aimed to examine the individual socio-demographic and household factors associated with the use of insecticide treated net by pregnant women in Ghana. The results indicate that the use of ITN among pregnant women in Ghana is low, with only 4 out of 10 pregnant women reporting the use of an ITN the previous night before the survey to prevent malaria. The individual socio-demographic factors (model I) associated with pregnant women’s use of ITN are their educational level and place of residence. The household factors (model II) influencing pregnant women’s use of ITNs for malaria prevention are the sex and age of household heads. A combination of individual socio-demographic and household factors in model III revealed age, and ethnic background of pregnant women, and household level factors such as sex and age of household heads and household wealth quintile were significant in predicting ITN use among pregnant women in Ghana.

### Synthesis with earlier studies

The prevalence of ITN use by pregnant women in this study is similar to prevalence reported in other sub-Saharan African countries such as Ethiopia (47.6%) [34], Kenya (52%) [39], Malawi (45.9) [32], Nigeria (19.2%) [40] and Uganda (35%) [42]. Reasons associated with the relatively low utilisation of ITN by pregnant women in these studies were high room temperature resulting in discomfort [20, 33, 37, 38], belief that malaria is no longer a major health problem [29, 43], poor quality of ITNs [44], reliance on other alternative malaria preventive measures [45]. The predominant reason for not using ITN by pregnant women is the discomfort experienced when sleeping under these treated bednets due to the high room temperature. Studies within the Africa and Asia regions suggest that treated bednet reduces the flow of air [46], and this explains the heat and warmness under a bednet [47–49]. There have been attempts by studies on a pilot basis to include small fans to increase the flow of air with the aim of ensuring comfort under treated bednets, thereby increasing the proportion of pregnant women sleeping under treated bed net [50]. This is important as several building in Ghana are poorly ventilated in additional to poor layout plans. Consequently, affecting room temperature and use of ITN.

The study showed that pregnant women aged 35–49 years had higher odds of using ITN to prevent malaria, compared to those aged 15–24 years after controlling for household factors. The finding of this study supports results of earlier studies in Cameroon [37], Kenya [39] and Senegal [51], which found increasing ITN use associated with relatively older pregnant women. However, a number of studies have found contrary evidence where higher ITN use was found among relatively younger pregnant women (20 years and below) relative to older women (30 years and above) [33, 34, 36, 52]. Other studies found no significant association between age of pregnant women and ITN utilisation [32, 35, 38, 43].

This study also found that pregnant women with no formal education and those who attained secondary education had higher odds of using ITN to prevent malaria, compared to those who had attained tertiary education without controlling for the effect of household factors. This finding is contrary to earlier studies carried out in sub-Saharan Africa, which found higher ITN use among highly educated pregnant women compared to uneducated pregnant women [20, 34, 37, 38]. The probable dominant reason given is that people with higher education are expected to have deeper knowledge and understanding about the usefulness and essence of using ITN for malaria prevention. This study reveals how the households’ pregnant women belong to influence their individual factors in ITN utilization in malaria prevention. For instance, ITN use is generally lower among uneducated pregnant women in the literature, however, the result of this study shows higher use of ITN among uneducated pregnant women relative to the educated ones after controlling for the effects of their household characteristics.

The study also showed that pregnant women residing in urban areas had lower odds of using ITN for malaria prevention relative to women in the rural areas. This study finding seems contrary to the findings of previous studies in the African sub-region that found higher use of ITN among pregnant women residing in urban areas [22, 32, 38, 53, 54]. The predominant reasons attributed to this phenomenon is the possibility of pregnant women having access to malaria prevention resources including ITN, pregnant women in urban centres having more knowledge about the essence of malaria prevention especially during pregnancy [32] and pregnant women in the urban centre having more extensive media exposure than their rural counterparts [53]. The findings of this study however suggest that the rural-urban disparity in ITN use among pregnant women in malaria prevention needs to be relooked at. It is argued in the literature that there is high use of ITN in rural areas because of the existence of various malaria control efforts and free distribution of ITNs [35]. Also, rural residents are less able to afford malaria treatment and therefore take preventive measures more seriously than those residing in the urban areas [37].

Another important finding in this study is that pregnant women who belong to households headed by males were more likely to use ITN compared to female households. This is in line with other studies in Nigeria [54], Sierra Leone [17] and Liberia [55]. The basic explanation could be that male headed household are often characterised by display of strict rules, discipline, and authority in making household decision. This may influence the adherence and regular use of ITN by pregnant women in such households [17].

Age of household head was also significant in predicting ITN use among pregnant women for malaria prevention. The odds of pregnant women using ITN is lower in households with relatively older (30–59) heads compared to heads of household aged 20–29 years. This study findings corroborates with an earlier study in Kenya [39]. This implies that as the age of household head gets increases, the odds of using an ITN decreases. It was also found that pregnant women who belong to the Akan ethnic group had higher odds of using ITN for malaria prevention, compared to pregnant women in other ethnic groups. Other related studies in Ghana did not find any significant relationship between ethnicity and ITN use among pregnant women [56].

### Strengths and limitations of the study

The main strength of this study is the use of malaria-related nationally representative data to examine individual and household factors associated with the use of ITN among pregnant women in Ghana. The findings can, therefore, be generalised to all pregnant women in Ghana. Regardless of these outlined strengths, this was a cross sectional study, and it will be difficult to deduce any causal interpretation. Also, the use of ITN may be influenced by seasonality of mosquito abundance and the data did not take into consideration this phenomenon. However, this we believe did not to a large extent affect the accuracy of result obtained. Finally, because the study used secondary data, it could not account for other factors at the community and national level that might have influenced pregnant women’s use of ITNs.

## Conclusion

The study found that 49.2% of pregnant women use ITN for malaria prevention in Ghana. Both individual and household factors were related to use of ITN among pregnant women. Particularly, age, educational level, place of residence, ethnicity, sex and age of household head and household wealth index were related to ITN utilisation among pregnant women. These factors ought to be reconsidered to improve and strengthen various malaria prevention strategies among vulnerable populations and in malaria endemic areas by the National Malaria Control Programme in achieving universal coverage of ITN among pregnant women.

## Data Availability

The datasets used for this study is openly available and can be accessed via https://dhsprogram.com/.

## Abbreviations

AOR: Adjusted Odds Ratio
CI: Confidence Interval
DHS: Demographic and Health Surveys
GPS: Global Positioning System
GMIS: Ghana Malaria Indicator Survey
GSS: Ghana Statistical Service
iCCM: Integrated Community Case Management
ICF: Inner City Fund
IPTp: Intermittent Preventive Treatment in Pregnancy
IRB: Institutional Review Board
IRS: Indoor Residual Spraying
ITN: Insecticides Treated Bed net
JMP: Joint Monitoring Programme
LPG: Liquified Petroleum Gas
LLIN: Long Lasting Insecticide Net
MIP: Malaria In Pregnancy
MOH: Ministry of Health
NMCP: National Malaria Control Programme
SMC: Seasonal Malaria Chemoprevention
UNICEF: United Nation International Children Emergency Fund
VIP: Ventilated Improved Pit
WHO: World Health Organisation
